# Sequencing the Barcelona‐MRI predictive model and Proclarix for improving the uncertain PI‐RADS 3

**DOI:** 10.1002/bco2.448

**Published:** 2024-10-22

**Authors:** Juan Morote, Ana Celma, Olga Méndez, Enrique Trilla

**Affiliations:** ^1^ Department of Surgery Universitat Autònoma de Barcelona Bellaterra Spain; ^2^ Department of Urology Vall d'Hebron University Hospital Barcelona Spain; ^3^ Biomedical Research Group in Urology Vall d'Hebron Research Institute Barcelona Spain

**Keywords:** Barcelona‐MRI PM, PI‐RADS, Proclarix, prostate cancer, screening

Risk‐stratified screening of prostate cancer (PCa) is currently recommended by the European Union (EU). Serum prostate‐specific antigen (PSA) testing is used to identify men suspected of having PCa, while magnetic resonance imaging (MRI) is used to select candidates for prostate biopsy.^1^ Prostate Imaging‐Reporting and Data System (PI‐RADS) is a score used to identify lesions suspected of having significant PCa (sPCa) on MRI. Prostate biopsies are typically avoided in men with PI‐RADS lesions 1 and 2 (negative MRI), as their negative predictive value reaches 97%. Prostate biopsy is recommended in men with PI‐RADS 3 to 5, with PI‐RADS 3 being the most uncertain scenario,^2^ as its positive predictive value for sPCa is between 16% and 18% with an overall 95% confidence interval (CI) between 13% and 27%.^3^, ^4^ To improve the selection of candidates for prostate biopsy in uncertain scenarios, the use of PSA density (PSAD), appropriate predictive models (PMs) and modern tumour markers is recommended.^5^

The European Association of Urology (EAU) currently recommends designing useful pathways that sequence stratifications based on appropriate PMs for men suspected of having PCa before and after MRI, with the objective of improving the efficacy of PCa screening by reducing MRI demand, prostate biopsies and the over‐detection of insignificant PCa (iPCa).^1^ The Barcelona (BCN) risk‐organized model, which stratifies men suspected of having PCa through the BCN‐PMs one (before MRI) and two (after MRI), has enhanced of the efficacy of detecting sPCa.^6^, ^7^ The BCN‐MRI PM has exhibited higher efficacy than PSAD for selecting men for prostate biopsy, especially in those with PI‐RADS 3.^8^ On the other hand, Proclarix, a new tumour marker that combine serum measurements of thrombospondin, cathepsin and percent free PSA, along with age, has shown good performance for detecting sPCa improving on that observed with PSAD and the Rotterdam‐MRI PM. Proclarix has been able to achieve a 100% sensitivity for sPCa within men with PI‐RADS 3.^8^

Since the BCN‐MRI PM and Proclarix have shown individually good performances for selecting candidates for prostate biopsy in men with PI‐RADS 3, we aim to demonstrate if their sequential use improves the selection of candidates for prostate biopsy.

We have conducted a head‐to head analysis of the BCN‐MRI PM and Proclarix in 169 men with serum PSA level above 3 ng/mL and/or suspicious digital rectal examination (DRE), and PI‐RADS v.2 score 3, consecutively referred from the opportunistic sPCa screening programme of Catalonia, Spain, between January 2018 and March 2019 at one academic institution. All participants underwent 2‐ to 4‐core transrectal MRI‐ultrasound fusion targeted biopsies and 12‐core systematic biopsies. Blood was obtained just before prostate biopsy, and 2 cc aliquots of serum stored at −80°C were sent to Proteomedix Inc. (Zurich‐Schlieren, Switzerland) in March 2020 for Proclarix assessment. The probability of sPCa, based on the BCN‐MRI PM, was assessed using the web risk calculator available at https://mripcaprediction.shinyapps.io/MRIPCaPrediction/ (last assessment on 23 April 2024). The project was approved by the institutional ethics committee (PR‐AG129/2020).

Men in the study cohort had a median age of 66 years (interquartile range [IQR] 60–72), a median serum PSA of 6 ng/mL (IQR 3.6–10.2) and a median prostate volume of 66 mL (IQR 45–85). A 71.6% of men were biopsy naïve, 6.5% had first degree PCa family history and 7.1% presented with suspicious DRE. Overall PCa was detected in 53 men (31.4%), with 25 of them (14.8%) having sPCa (grade group ≥2), and 28 (16.6%) having iPCa. The areas under the curve and (95% CI) of the BCN‐MRI PM and Proclarix were 0.797 (0.711–0.883), and 0.702 (0.615–0.789), respectively (*p* < 0.001). The thresholds of the BCN‐MRI PM and Proclarix for detecting 100% of sPCa were of 6.8% and 10%, respectively, and the corresponding specificities were 41.7% and 25%, respectively (*p* < 0.001). The positive predictive values of the BCN‐MRI and Proclarix were 22.9% and 18.8%, respectively, and their negative predictive value was100%. The efficacy of BCN‐MRI PM for detecting sPCa was 50.3%, compared to 36.1% for Proclarix. The BCN‐MRI PM avoided prostate biopsies in 60 men (35.5%) while Proclarix avoided them in 36 men (21.3%) (*p* < 0.001). Over‐detection of iPCa was avoided in seven cases (4.1%) with each of the BCN‐MRI PM Proclarix.

The BCN‐MRI PM exhibited better performance than Proclarix for enhancing the efficacy of sPCa detection in men with PI‐RADS 3. However, to explore the complementarity of both tools we investigated the effectiveness of a pathway sequencing the stratification of men first with the BCN‐MRI PM, and secondly with Proclarix in those men with a probability of sPCa higher than 6.8%. The rationale for this order was the better performance of the BCN‐MRI PM and the fact that is free. Results of applying this pathway are presented in Figure 1. We note, in the first stratification using the BCN‐MRI‐PM, that prostate biopsy was avoided in 60 cases (35.5%) where the sPCa risk was 6.8% or less, and the over‐detection of iPCa decreased in seven cases (4.1%). A second stratification, based on Proclarix, was conducted in 109 men (64.4%) with a BCN‐MRI PM higher than 6.6%. Proclarix avoided 17 prostate biopsies (10.1%) in men with a risk of sPCa 10% or less, in which five iPCas were undetected. Among the 77 men in whom prostate biopsy was avoided (45.6%) after applying this sequenced pathway, no sPCa was found while 12 of 28 iPCas (42.9%) were undetected. Finally, 92 men with Proclarix higher than 10% (54.4%) underwent prostate biopsy, detecting all 25 sPCas, and 16 iPCas (57.1%). The efficacy of prostate biopsy for detecting sPCa improved from 14.8% when all PI‐RADS 3 cases were biopsied to 27.1% when the sequential pathway was applied, resulting in an 83.1% increase in prostate biopsy efficacy. In summary, this sequential pathway, applied in men with PI‐RADS 3, would be able to detect all sPCas, avoid 45.6% of prostate biopsies and reduce the over‐detection of iPCa in 42.9%, increasing the efficacy of prostate biopsy by 83.1%.

**FIGURE 1:**
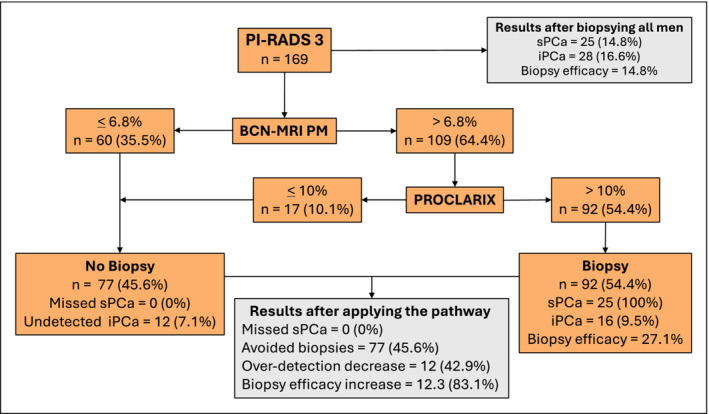
Pathway sequencing the Barcelona‐magnetic resonance imaging (BCN‐MRI) predictive model (PM) and Proclarix for improving significant prostate cancer (sPCa) detection in men with Prostate Imaging‐Reporting and Data System (PI‐RADS) 3. Biopsy efficacy = sPCa / number of biopsies. iPCA; insignificant PCa.

The BCN‐MRI PM and Proclarix have both exhibited optimal sensitivity, since they were able to detect all sPCa cases. The BCN‐MRI PM overperformed Proclarix. However, the most interesting finding was that Proclarix improved the results obtained with the BCN‐MRI PM when it was measured in men with a probability of sPCa higher than 6.8%. Proclarix needed to be assessed in 64.4% men with PI‐RADS 3. A simple cost‐effectiveness analysis shows that $92 400 (77 avoided prostate biopsies at $1200 each) will be saved with an expenditure of $27 600 (92 assessments of Proclarix at $300 each).

The strength of this study is the head‐to head design, which allowed for a realistic stratification based on sequencing men with the BCN‐MRI PM and Proclarix. Limitations included the unicentric design and small size of the analysed cohort. The definition of sPCa in prostate biopsy does not represent true incidence of sPCa in the whole prostate gland. The storage of frozen serum until Proclarix assessment could represent a measurement bias.

Future randomized and multicentric trials should confirm the promising results obtained from sequencing stratifications with the BCN‐MRI PM and Proclarix in men suspected of having PCa with PI‐RADS 3.

## AUTHOR CONTRIBUTIONS

Juan Morote conceptualized the idea. Ana Celma and Olga Méndez and Enrique Trilla developed the concept. Juan Morote wrote the first draft of the manuscript. All authors were involved in editing, critical review and final approval of the manuscript.

## CONFLICT OF INTEREST STATEMENT

The authors have no conflict of interest to declare.
